# 1,25-Dihydroxyvitamin D and the Vitamin D Receptor Gene Polymorphism Apa1 Influence Bone Mineral Density in Primary Hyperparathyroidism

**DOI:** 10.1371/journal.pone.0056019

**Published:** 2013-02-13

**Authors:** Monika H. E. Christensen, Ellen M. Apalset, Yngve Nordbø, Jan Erik Varhaug, Gunnar Mellgren, Ernst A. Lien

**Affiliations:** 1 Institute of Medicine, University of Bergen, Bergen, Norway; 2 Hormone Laboratory, Haukeland University Hospital, Bergen, Norway; 3 Department of Rheumatology, Haukeland University Hospital, Bergen, Norway; 4 Department of Public Health and Primary Health Care, Research Group for Lifestyle Epidemiology, University of Bergen, Bergen, Norway; 5 Department of Surgery, Haukeland University Hospital, Bergen, Norway; 6 Department of Surgical Science, University of Bergen, Bergen, Norway; University of Tennessee, United States of America

## Abstract

**Objective:**

Parathyroid hormone (PTH) and vitamin D are the most important hormones regulating calcium metabolism. In primary hyperparathyroidism (PHPT) excessive amounts of PTH are produced. Bone turnover is enhanced, leading to reduced bone mineral density and elevated levels of serum calcium. The aim of this study was to investigate relations between serum levels of 25-hydroxyvitamin D (25(OH)D), 1,25-dihydroxyvitamin D (1,25(OH)_2_D) and bone mineral density, as well as known genetic polymorphisms in the vitamin D receptor and enzymes metabolising vitamin D in patients with PHPT.

**Design/Subjects:**

We conducted a cross-sectional study of 52 patients with PHPT.

**Results:**

Mean level of 25(OH)D was 58.2 nmol/L and median 1,25(OH)_2_D level was 157 pmol/L. Among our patients with PHPT 36.5% had 25(OH)D levels below 50 nmol/L. Serum 1,25(OH)_2_D was inversely correlated to bone mineral density in distal radius (p = 0.002), but not to bone mineral density at lumbar spine or femoral neck. The vitamin D receptor polymorphism Apa1 (rs7975232) was associated with bone mineral density in the lumbar spine.

**Conclusions:**

The results suggest that PHPT patients with high blood concentrations of 1,25(OH)_2_D may have the most deleterious skeletal effects. Randomized, prospective studies are necessary to elucidate whether vitamin D supplementation additionally increases serum 1,25(OH)_2_D and possibly enhances the adverse effects on the skeleton in patients with PHPT.

## Introduction

Primary hyperparathyroidism (PHPT) is characterized by elevated serum levels of parathyroid hormone (PTH) and calcium. In most cases PHPT is caused by a benign adenoma or glandular hyperplasia producing excessive amounts of PTH [1]. Elevated levels of PTH increase calcium resorption in the kidneys, leading to hyper-calcemia, while phosphate excretion is enhanced. Bone turnover is enhanced, reducing bone mineral density (BMD) and elevating levels of bone markers [2]. Increased fracture risk is described in PHPT patients [3].

**Figure 1:**
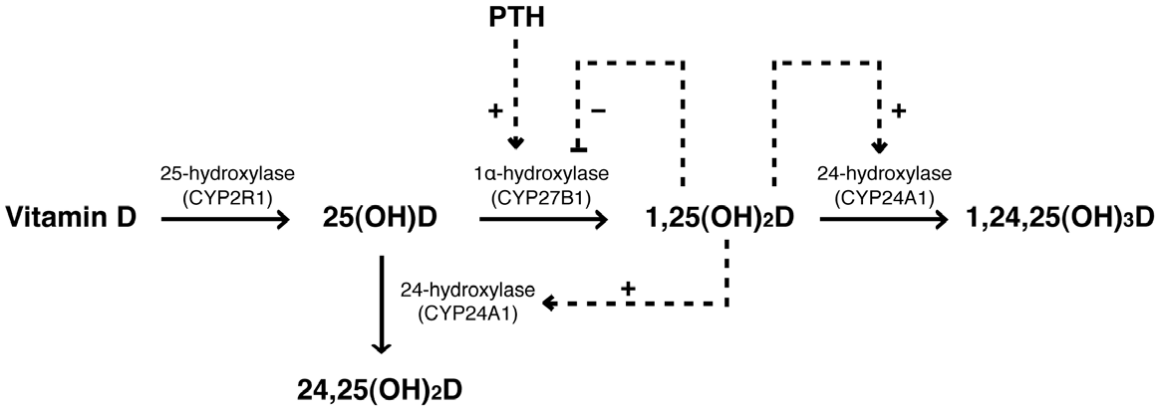
Vitamin D metabolism. 1,25(OH)_2_D and PTH influence vitamin D metabolism by positive (+) or negative (−) regulation of the activity of the 1α-hydroxylase and the 24-hydroxylase. 25(OH)D, 25-hydroxyvitamin D; 1,25(OH)_2_D, 1,25-dihydroxyvitamin D; 24,25(OH)_2_D, 24,25-dihydroxyvitamin D; 1,24,25(OH)_3_D, 1,24,25-trihydroxyvitamin D.

To become metabolically active, vitamin D has to be hydroxylated by cytochrome P450 enzymes (Figure 1). The major circulating form of vitamin D, 25-hydroxyvitamin D (25(OH)D), is formed in the liver. 25(OH)D is the most commonly used indicator of vitamin D status, reflecting the storage of the vitamin [4]. Further hydroxylation to 1,25-dihydroxyvitamin D (1,25(OH)_2_D) is catalysed by the 1α-hydroxylase. This enzyme is mainly localised in the kidneys but is also found in other tissues [5]. The activity of the 1α-hydroxylase is controlled by PTH and 1,25(OH)_2_D keeping serum levels of 1,25(OH)_2_D fairly constant [6], [7]. In PHPT-patients, high levels of PTH increase the activity of 1α-hydroxylase. Vitamin D inadequacy seems to be more frequent in PHPT patients than in the background population [8], [9]. Low levels of 25(OH)D in these patients may be explained through increased activity of both the 1α-hydroxylase and the 24-hydroxylase [7], [10], [11].

Environmental factors like exposure to sunlight, nutrition, and seasonal variation influence vitamin D status [12], [13], [14], though, individual variations in vitamin D-concentrations are not fully explained by external factors. Twin studies have shown individual differences in bone formation, bone resorption, calcium homeostasis, concentrations of PTH and vitamin D due to genetic variation [15]. Influences of single nucleotide polymorphisms (SNPs) are independent of lifestyle and are life long. Differences in BMD, vitamin D-metabolism and calcium levels in patients with PHPT could be explained by genetic variation in enzymes metabolising vitamin D. Furthermore, 1,25(OH)_2_D is a ligand for the intra-nuclear vitamin D receptor (VDR) that regulates gene transcription of vitamin D responsive genes [16]. Accordingly, polymorphisms in the VDR-gene may have an influence on vitamin D-sensitive tissues, such as bone [17].

The aim of this study was to investigate serum levels of 25(OH)D and 1,25(OH)_2_D and the influence of these vitamin D metabolites on BMD in patients with PHPT. We also investigated whether common SNPs in enzymes important in the vitamin D metabolism and the VDR could explain individual differences in disease severity, especially regarding BMD. We observed an inverse correlation between BMD in distal radius and concentrations of 1,25(OH)_2_D. Additionally, we found that BMD in lumbar spine was associated with the Apa1 polymorphism in the VDR gene.

## Materials and Methods

### Ethics Statement

The study was performed according to the principles expressed in the declaration of Helsinki. All enrolled subjects signed an informed written consent. The Western Norway Regional Committee for Medical Research Ethics (REK) approved the study.

### Subjects and Study Design

52 patients (42 females and 10 males) scheduled to undergo surgery for PHPT participated in the study. Patients included had PHPT caused by either single adenomas (85%) or multi-glandular hyperplasia (15%). PHPT was histologically confirmed in all patients. The diagnosis of PHPT was based on serum levels of ionised calcium above the reference range (reference range: 1.15–1.30 mmol/L) and PTH-levels that were elevated or in the upper third of the reference range (reference range: 1.3–6.8 pmol/L). The recruitment period was from September 2007 to May 2010. Inclusion was consecutive and equally distributed throughout the year. Exclusion criteria were known inflammatory bowel disease, rheumatoid disease or lack of BMD-measurements. Patients with genetically confirmed multiple endocrine neoplasia type 1 (MEN-1 syndrom) or type 2a (MEN-2a syndrome) based on verified mutations in the *MENI* gene or *RET* gene, respectively, were excluded. During the study period, all included patients lived in the Western coastal part of Norway and underwent surgery at the Department of Endocrine Surgery, Haukeland University Hospital, Bergen, Norway. Blood samples, anthropometric data and medical history were collected the day before parathyroidectomy. BMD was measured within 4 months prior to surgery.

### Biochemical Analysis and Anthropometric Data

Blood samples from patients were centrifuged and frozen at -80°C within 2 hours after collection. Ionised calcium, albumin, phosphate, alkaline phosphatase (ALP), alanine transaminase (ALAT), and C-reactive protein (CRP) were analysed immediately at the Laboratory of Clinical Biochemisty, Haukeland University Hospital, Bergen, Norway, using the Modular P-system from Roche Diagnostics, Basel, Switzerland. PTH was measured at the Hormone Laboratory, Haukeland University Hospital, Bergen, Norway, using a two-site chemiluminescent immunometric assay for intact PTH (Immulite 2000, Siemens Healthcare Diagnostics, Deerfield, IL, USA). The inter-assay variations were 6.3% at a concentration of 5.6 pmol/L and 8.8% at 40 pmol/L. Plasma concentrations of 25(OH)D were analysed with a liquid chromatography/tandem mass spectrometry (LC-MS/MS) method developed at the Hormone Laboratory, Haukeland University Hospital, Bergen, Norway [18]. 25(OH)D_3_ and 25(OH)D_2_ were measured separately using this method. A radioimmunoassay kit from Immunodiagnostic system, Boldon, UK, was used for analysing 1,25(OH)_2_D. This assay had a sensitivity of 100% for 1,25(OH)_2_D_3_ and 91% for 1,25(OH)_2_D_2_. Detection limit was 8 pmol/L for 1,25(OH)_2_D and the inter-assay variations were 13.6%, 9.6% and 9.6% for concentrations of 20.5 pmol/L, 60.8 pmol/L and 135.2 pmol/L, respectively. Creatinine was determined by including it and its deuterated internal standard (d3-creatinine) in an established LC-MS/MS assay using the ion pairs 114/44.2 and 117/47.2, respectively [19]. Creatinine was analysed at Bevital A/S, Bergen, Norway. The isotope dilution mass spectrometry (IDMS) traceable formula developed by the Modification of Diet in Renal Disease (MDRD) Study Group was used for estimated glomerular filtration rate (eGFR) calculations: eGFR = 175×(s-creatinine/88.4)^ –1.154^×(Age)^–0.203^×0.742 if female [20]. Body mass index (BMI) was calculated as the weight divided through the square of the body length (kg/m^2^).

### Bone Mineral Density

BMD was measured at the Department of Rheumatology, Haukeland University Hospital, Bergen, Norway, using a stationary, dual energy X-ray absorptiometry (DXA) (Lunar Prodigy, GE Healthcare, Diegem, Belgium). Calibration of the scanner was performed against a standard calibration block each day, and was stable during the whole measurement period. The *in vitro* long-term coefficient of variation was 0.86%, and the *in vivo* short-term precision for femoral neck was 1.47%. BMD in distal radius, lumbar spine (L2–L4) and femoral neck was available for 52 patients and BMD was measured within four months prior to surgery. Left distal radius was measured in all except four patients, where right radius was measured. Right femoral neck was used in all cases.

### Purification of DNA

Genomic leucocyte DNA was purified from EDTA-anticoagulated whole blood using the MagNa PURE LC instrument and the MagNa PURE LC DNA isolation kit (Roche Diagnostics, GmbH, Mannheim, Germany). Quality and concentration of DNA were measured using the NanoDrop® ND-100 spectrophotometer (NanoDrop Technologies, Wilmington, USA).

### Single Nucleotide Polymorphisms

Based on previous studies we selected 10 SNPs located in genes coding for 25-hydroxylase/CYP2R1 (rs10741657 and rs2060793), 24-hydroxylase/CYP24A1 (rs6013897 and rs2762939), 1α-hydroxylase/CYP27B1 (rs10877012 and rs703895) and VDR (rs1544410 (Bsm1), rs731236 (Taq1), rs7975232 (Apa1), and rs2228570 (Fok1)) [21], [22], [23], [24], [25]. The nucleic acid sequences of the selected SNPs were found through the database http://snpper.chip.org/. SNPs and alleles were verified using the database http://hapmap.ncbi.nlm.nih.gov/. Kbioscience, Hoddesdon, UK, analysed the selected SNPs from purified DNA, using the Allele-Specific Polymorphism (KASP) genotyping system (http://www.kbioscience.co.uk).

### Statistical Analysis

Continuous variables are reported as mean (standard deviation) or median (25^th^ to 75^th^ percentile) and categorical variables as counts (percentages). PTH, ALP, ALAT, and 1,25(OH)_2_D did not show normal distribution and values were logarithmically transformed before used in statistical tests. Differences across the tertiles of 25(OH)D, 1,25(OH)_2_D and PTH were calculated using linear regression. Age, gender and BMI were included as covariates when analysing BMD in distal radius, lumbar spine and femoral neck. For differences across the tertiles of 25(OH)D, age and BMI were covariates as these differed between the 25(OH)D-tertiles. To assess differences in gender across the tertiles, a chi-square test was used. Additionally, multiple linear regression was performed with BMD in distal radius, lumbar spine and femoral neck as the dependent variable, using age, BMI, gender, phosphate, eGFR, ionised calcium, 25(OH)D, 1,25(OH)_2_D, and PTH as covariates. Time of blood sampling was grouped using categorical variables for winter (January to March), spring (April to June), summer (July to September) and autumn (October to December). Season was used as a covariate in the linear regression model. Correlations were assessed by Spearman’s rho test. Associations between SNP-variants and BMD at the forearm, lumbar spine and right femoral neck were assessed using linear regression. These tests were performed once assuming that minor allele was dominant and once assuming that minor allele was recessive. All analysis including BMD in distal radius, lumbar spine and femoral neck were adjusted for age, gender and BMI, as these parameters are strong predictors of BMD [26], [27]. All tests were two-sided and p<0.05 was considered statistically significant. In the SNP-analysis, correction for multiple testing was performed using the Bonferroni method. Thus, where multiple SNPs were analysed a correction factor of 7 (7 SNPs included in the analyses) was used. After Bonferroni-correction a p-value <0.007 was required for statistical significance. Statistical analyses were performed using SPSS Statistics 19 for Mac (IBM Corporation, New York, USA).

Linkage disequilibrium for the selected genes was checked with the free internet-based program haploview (version 2, release 21, www.broad.mit.edu/mpg/haploview/). Calculations of Hardy Weinberg equilibrium for the tested SNPs were based on a chi-squared test, assessed with the internet-based software PLINK (version 1.0, http://pngu.mgh.harvard.edu/purcell/plink/) [28].

## Results

### Subject Characteristics

Characteristics of the included 52 patients are listed in table 1. 25(OH)D levels below 50 nmol/L were observed in 36.5% of the patients. Median 1,25(OH)_2_D-level was 157 pmol/L, which is above the reference range (50–150 pmol/L). None of the patients had detectable serum level of 25(OH)D_2_ and none of the patients used vitamin D supplements prescribed by a physician. Seven patients had eGFR under 60 mL/min/1.73m^2^ (reference range: >60 mL/min/1.73m^2^).

**Table 1:** Patient characteristics at inclusion (n = 52).

Characteristics		
**Females** (%)^a^	42	(80.8)
**Age** (years)	60.8	(12.7)
**25(OH)D** (nmol/L)	58.2	(21.5)
**1,25(OH)_2_D** (pmol/L)	157	(130–197.5)
**BMI** (kg/m^2^)	26.4	(4.20)
**PTH** (pmol/L)	13.1	(9.63–18.5)
**iCa** (mmol/L)	1.48	(0.092)
**Phosphate** (mmol/L)	0.84	(0.17)
**Albumin** (g/L)	45.6	(2.66)
**ALP** (U/L)	85.5	(73.3–117)
**eGFR** (mL/min/1.73 m^2^)	84.0	(22.1)
**ALAT** (U/L)	26.0	(18.3–31.8)
**CRP** (mg/L)	1.0	(0.10–2.0)

Values are given as mean (SD) or median (25^th^ to 75^th^ percentile). PHPT, primary hyperparathyroidism; BMI, body-mass index; PTH, parathyroid hormone; iCa, ionised calcium; APL, alkaline phosphatase; eGFR, estimated glomerular filtration rate; ALAT, alanine transaminase; CRP, C-reactive protein; 25(OH)D, 25-hydroxyvitamin D; 1,25(OH)_2_D, 1,25-dihydroxyvitamin D.

a Values are numbers (percentages).

### Relationship between 25(OH)D, 1,25(OH)_2_D, PTH and Bone Mineral Density

Patients were divided into tertiles according to levels of 25(OH)D (Table 2), 1,25(OH)_2_D (Table 3) and PTH (Table 4). Radial BMD decreased significantly across increasing tertiles of 1,25(OH)_2_D and PTH. 1,25(OH)_2_D increased with increasing PTH. eGFR differed significantly across the tertiles of 1,25(OH)_2_D, with lowest levels in the group with lowest 1,25(OH)_2_D concentrations. No differences in albumin, ALAT and CRP were found according to tertiles of either 25(OH)D, 1,25(OH)_2_D or PTH (data not shown). When analysing a sub-group including only the 45 patients with eGFR >60 mL/min/1.73m^2^, age did not differ between the tertiles of 25(OH)D (p = 0.12), and eGFR did not differ between the tertiles of 1,25(OH)_2_D (p = 0.073). Examining only these patients did not change the results regarding differences in BMD in distal radius, lumbar spine and femoral neck across any of the tertiles compared to analysing all 52 patients. We therefore decided to include all patients in the further analyses. Additional adjustment for season when blood samples were drawn did not change any of the results (data not shown).

**Table 2:** Patients divided into tertiles according to serum levels of 25(OH)D.

	Tertiles of 25(OH)D							
	Range: 22–47 nmol/L (n = 17)		Range: 47–65 nmol/L (n = 18)		Range: 67–115 nmol/L (n = 17)		p-value	
Females (%)^a^	13	(76.5)	16	(88.9)	13	(76.5)	0.56	
Age	55.9	(14.7)	61.4	(11.3)	64.9	(10.8)	0.038	
BMI (kg/m^2^)	28.0	(4.12)	26.1	(3.47)	24.9	(4.47)	0.013^b^	
1,25(OH)_2_D (pmol/L)	151	(124–205)	138	(122–198)	172	(146–193)	0.32^c^	
PTH (pmol/L)	15.0	(8.55–23.4)	12.8	(9.55–18.9)	12.7	(10.4–16.6)	0.28^c^	
iCa (mmol/L)	1.52	(0.09)	1.47	(0.09)	1.44	(0.09)	0.069^c^	
Phosphate (mmol/L)	0.81	(0.15)	0.90	(0.20)	0.80	(0.13)	0.23^c^	
eGFR (mL/min/1.73 m^2^)	91.7	(19.8)	83.0	(22.4)	77.4	(22.9)	0.096^c^	
ALP (U/L)	94.0	(75.5–124)	89.0	(68.5–108)	86.0	(72.5–114)	0.35^c^	
BMD radius (g/cm^2^)	0.50	(0.11)	0.46	(0.11)	0.45	(0.10)	0.55^d^	
BMD lumbal spine (g/cm^2^)	1.12	(0.17)	1.03	(0.19)	1.06	0.19)	0.42^d^	
BMD femural neck (g/cm^2^)	0.86	(0.13)	0.77	(0.12)	0.79	(0.10)	0.62^d^	

Values are given as mean (standard deviation) or median (25th–75th percentile). PTH, 1,25(OH)_2_D and ALP were log-transformed before used in the analyses. BMI, body mass index; PHPT, primary hyperparathyroidism; PTH, parathyroid hormone; iCa, ionised calcium; APL, alkaline phosphatase; 25(OH)D, 25-hydroxyvitamin D; 1,25(OH)_2_D, 1,25-dihydroxyvitamin D; eGFR, estimated glomerular filtration rate; BMD, bone mineral density. P-values for linear trend over tertiles.

a Values are numbers (percentages), differences across tertiles are assessed with a chi-square test.

b Age was used as covariate.

c Age and BMI were used as covariates.

d Age, BMI and gender were used as covariates.

**Table 3:** Patients divided into tertiles according to serum levels of 1,25(OH)_2_D.

	Tertiles of 1,25(OH)_2_D								
		Range: 48–137 pmol/L n = 17		Range: 138–176 pmol/L n = 18		Range: 179–350 pmol/L n = 17		p-value	
Females (%)^a^		13	(76.4)	14	(77.8)	15	(88.2)	0.63	
Age		60.5	(12.3)	61.1	(10.6)	60.6	(15.5)	0.99	
BMI (kg/m^2^)		26.1	(3.66)	26.8	(5.09)	26.2	(3.79)	0.96	
25(OH)D (nmol/L)		47.7	(15.7)	67.6	(21.2)	58.6	(23.2)	0.14	
PTH (pmol/L)		11.4	(8.70–15.2)	11.2	(8.60–14.1)	18.2	(12.5–24.8)	0.011	
iCa (mmol/L)		1.48	(0.09)	1.46	(0.08)	1.51	(0.10)	0.29	
Phosphate (mmol/L)		0.90	(0.17)	0.83	(0.13)	0.80	(0.18)	0.062	
eGFR (mL/min/1.73 m^2^)		76.8	(21.3)	81.9	(20.7)	93.3	(22.3)	0.029	
ALP (U/L)		80.0	(70.0–97.0)	99.0	(73.3–124)	89.0	(79.5–125)	0.30	
BMD radius (g/cm^2^)		0.48	(0.11)	0.50	(0.80)	0.41	(0.11)	0.002^d^	
BMD lumbal spine (g/cm^2^)		1.04	(0.19)	1.09	(0.15)	1.07	(0.22)	0.66^d^	
BMD femural neck (g/cm^2^)		0.77	(0.12)	0.82	(0.10)	0.82	(0.14)	0.11^d^	

Values are given as mean (standard deviation) or median (25th–75th percentile). PTH, 1,25(OH)_2_D and ALP were log-transformed before used in the analyses. BMI, body mass index; PHPT, primary hyperparathyroidism; PTH, parathyroid hormone; iCa, ionised calcium; APL, alkaline phosphatase; 25(OH)D, 25-hydroxyvitamin D; 1,25(OH)_2_D, 1,25-dihydroxyvitamin D; eGFR, estimated glomerular filtration rate; BMD, bone mineral density. P-values for linear trend over tertiles.

a Values are numbers (percentages), differences across tertiles are assessed with a chi-square test.

d Age, BMI and gender were used as covariates.

**Table 4:** Patients divided into tertiles according to serum levels of PTH.

	Tertiles of PTH						
	Range: 5.6–10.5 pmol/L n = 17		Range: 10.8–15.8 pmol/L n = 18		Range: 17.5–51.3 pmol/L n = 17		p-value
Females (%)^a^	13	(76.4)	15	(83.3)	14	(82.4)	0.86
Age	59.8	(11.7)	60.7	(10.8)	61.8	(15.8)	0.65
BMI (kg/m^2^)	27.3	(4.41)	25.0	(3.58)	27.0	(4.50)	0.75
25(OH)D (nmol/L)	55.5	(18.1)	62.4	(23.4)	56.3	(23.2)	0.92
1,25(OH)_2_D (pmol/L)	140	(99.5–159)	159	(128–179)	204	(138–246)	<0.001
iCa (mmol/L)	1.43	(0.07)	1.47	(0.08)	1.55	(0.08)	<0.001
Phosphate (mmol/L)	0.90	(0.16)	0.85	(0.17)	0.77	(0.15)	0.017
eGFR (mL/min/1.73 m^2^)	87.1	(19.0)	81.9	(19.6)	83.1	(27.8)	0.598
ALP (U/L)	89.0	(69.0–105)	81.0	(72.5–123)	90.0	(81.5–127)	0.42
BMD radius (g/cm^2^)	0.50	(0.08)	0.47	(0.10)	0.43	(0.13)	0.027^d^
BMD lumbal spine (g/cm^2^)	1.06	(0.14)	1.09	(0.22)	1.05	(0.19)	0.67^d^
BMD femural neck (g/cm^2^)	0.81	(0.13)	0.80	(0.9)	0.80	(0.15)	0.19^d^

Values are given as mean (standard deviation) or median (25th–75th percentile). PTH, 1,25(OH)_2_D and ALP were log-transformed before used in the analyses. BMI, body mass index; PHPT, primary hyperparathyroidism; PTH, parathyroid hormone; iCa, ionised calcium; APL, alkaline phosphatase; 25(OH)D, 25-hydroxyvitamin D; 1,25(OH)_2_D, 1,25-dihydroxyvitamin D; eGFR, estimated glomerular filtration rate; BMD, bone mineral density. P-values for linear trend over tertiles.

a Values are numbers (percentages), differences across tertiles are assessed with a chi-square test.

d Age, BMI and gender were used as covariates.

In addition to studying variations across tertiles, we also analysed correlations between parameters using Spearman’s rho test, adjusted for age, gender and BMI when including BMD. We observed a negative correlation between BMD in distal radius and 1,25(OH)_2_D (r = −0.470, p = 0.001), while BMD in distal radius and PTH was not significantly correlated (r = −0.287, p = 0.062). PTH and 1,25(OH)_2_D were positively correlated (r = 0.412, p = 0.002) as were also 1,25(OH)_2_D and eGFR (r = 0.340, p = 0.014). We did not observe a correlation between 25(OH)D and 1,25(OH)_2_D, 25(OH)D and PTH, or 25(OH)D and BMD at any of the measured sites. ALP and iCa were positively correlated (r = 0.282, p = 0.043). Interestingly, 25(OH)D levels increased with age (r = 0.293, p = 0.035).

Multiple linear regression with age, BMI, gender, phosphate, ionised calcium, eGFR, 25(OH)D, 1,25(OH)_2_D, and PTH as covariates did not show an influence of 25(OH)D, 1,25(OH)_2_D or PTH on BMD in femoral neck and lumbar spine. 1,25(OH)_2_D was significantly associated with BMD in distal radius (standardised beta (effect size) = −0.28, p = 0.010). In this model PTH was not associated with BMD in distal radius (standardised beta = −0.024, p = 0.84). Age, gender, and BMI contributed significantly to differences in BMD in distal radius, whereas only age and BMI were associated with BMD in femoral neck and lumbar spine. Phosphate, ionised calcium, and eGFR did not contribute to differences in BMD at any of the investigated sites.

### Single Nucleotide Polymorphisms

None of the selected SNPs differed significantly from Hardy-Weinberg equilibrium. There was strong linking disequilibrium (LD) between several of the SNPs with gene location in the proximity of each other. Where LD was >0.8, only one of the SNPs was selected for further analysis. Rs10741657 on chromosome 11 in the 25-hydroxylase-gene was in LD with rs2060793 (r^2^ = 1.0). The SNP in the 1α-hydroxylase-gene, rs703842 on chromosome 12 was in LD with rs10877012 (r^2^ = 1.0). For the VDR-gene, rs1544410 localised on chromosome 12 was in LD with rs731236 (r^2^ = 0.96). 7 tag SNPs were selected for further analysis (Table 5). Several of the studied SNPs showed trends towards variation in BMD (Table 6). Only the SNP variant rs7975232 (Apa1) in the VDR showed a significant influence on BMD in lumbar spine after Bonferroni correction. When considering the minor allele (allele A) as dominant, we observed significant lower lumbar spine BMD in patients with genotype AA/CA vs. CC. We did not observe a relation between SNP genotypes and any of the biochemical parameters analysed (data not shown).

**Table 5:** Selected SNPs analysed in 52 individuals with primary hyperparathyroidism.

rs number	Genotype major/minor homozygote	Frequency major/minor homozygote	Included samples		HWE p-value		
***VDR***							
rs1544410 (Bsm1)	GG/AA	0.37/0.13	52		0.44		
rs7975232 (Apa1)	CC/AA	0.21/0.18	51		0.12		
rs2228570 (Fok1)	CC/TT	0.35/0.13	52		0.27		
***25-hydroxylase (Cyp2R1)***							
rs10741657	GG/AA	0.44/0.21	52		0.14		
***24-hydroxylase (Cyp24a1)***							
rs6013897	TT/AA	0.36/0.08	52	0.19			
rs2762939	GG/CC	0.46/0.06	52	0.21			
***1-alfa hydroxylase (Cyp27B1)***							
Rs10877012	GG/TT	0.53/0.12	51	0.25			

Only samples without missing values were included in the analyses. P-values for Hardy Weinberg equilibrium are based on a chi-squared test. SNP, single nucleotide polymorphism; HWE, Hardy Weinberg equilibrium; VDR, vitamin D receptor.

**Table 6:** Relations between the genotype variations of the SNPs studied and bone mineral density in lumbar spine, femoral neck and distal radius.

rs number		Genotype					Bone mineral density (g/cm^2^)								
					Frequency n (%)	Radius			Lumbar spine			Femoral neck			
***VDR***															
rs1544410		GG	19 (37)			0.48 (0.09)			1.14 (0.17)			0.85 (0.13)			
		GA	26 (50)			0.46 (0.12)			1.03 (0.19)			0.79 (0.12)			
		AA	7 (13)			0.46 (0.08)			1.00 (0.16)			0.72 (0.07)			
p-value^1^						0.39			0.046			0.036			
p-value^2^						0.88			0.41			0.065			
rs7975232		CC	11 (21)			0.50 (0.08)			1.21 (0.16)			0.87 (0.12)			
		CA	31 (61)			0.47 (0.12)			1.04 (0.17)			0.80 (0.13)			
		AA	9 (18)			0.44 (0.10)			1.01 (0.19)			0.74 (0.09)			
p-value^1^						0.21			0.003			0.032			
p-value^2^						0.79			0.47			0.26			
rs2228570		CC	18 (35)			0.47 (0.10)			1.05 (0.16)			0.80 (0.10)			
		CT	27 (52)			0.47 (0.11)			1.07 (0.21)			0.81 (0.15)			
		TT	7 (13)			0.48 (0.10)			1.10 (0.16)			0.79 (0.09)			
p-value^1^						0.35			0.49			0.95			
p-value^2^						0.87			0.57			0.70			
***25-hydroxylase (Cyp2R1)***															
rs10741657		GG	23 (44)			0.49 (0.11)			1.09 (0.17)			0.81 (0.13)			
		GA	18 (35)			0.48 (0.10)			1.11 (0.21)			0.83 (0.14)			
		AA	11 (21)			0.42 (0.10)			0.95 (0.12)			0.75 (0.08)			
p-value^1^						0.10			0.38			0.84			
p-value^2^						0.145			0.039			0.25			
***24-hydroxylase (Cyp24a1)***															
rs6013897	TT			19 (36)		0.50 (0.10)		1.15 (0.16)			0.82 (0.11)				
	TA			29 (56)		0.46 (0.10)				1.02 (0.18)			0.80 (0.11)		
	AA			4 (8)		0.42 (0.14)				1.03 (0.23)			0.78 (0.27)		
p-value^1^						0.26				0.018			0.83		
p-value^2^						0.40				0.83			0.86		
rs2762939	GG			24 (46)		0.48 (0.10)				1.05 (0.17)			0.80 (0.10)		
	GC			25 (48)		0.47 (0.11)				1.08 (0.21)			0.83 (0.14)		
	CC			3 (6)		0.42 (0.10)				1.04 (0.07)			0.67 (0.03)		
p-value^1^						0.74				0.57			0.60		
p-value^2^						0.52				0.99			0.13		
***1-alfa hydroxylase (Cyp27B1)***															
rs10877012	GG			27 (53)		0.46 (0.11)				1.09 (0.19)			0.79 (0.11)		
	GT			18 (35)		0.47 (0.10)				1.03 (0.17)			0.82 (0.14)		
	TT			6 (12)		0.49 (0.07)				1.05 (0.19)			0.80 (0.12)		
p-value^1^						0.77				0.24			0.95		
p-value^2^						0.52				0.97			0.46		

SNP, single nucleotide polymorphism; VDR, vitamin D receptor.

1 p-values are based on linear regression with age, gender and body mass index as covariates. Minor allele considered as dominant.

2 p-values are based on linear regression with age, gender and body mass index as covariates. Minor allele considered as recessive. After Bonferroni-correction a p-value <0.007 is considered to be statistically significant.

## Discussion

The main finding of the present study was the inverse correlation between serum 1,25(OH)_2_D and BMD in distal radius in patients with PHPT, indicating that high serum levels of 1,25(OH)_2_D may have deleterious skeletal effects. Moreover, we observed an association between BMD in the lumbar spine and the VDR polymorphism Apa1.

The observed inverse correlation between 1,25(OH)_2_D and BMD in distal radius is in accordance with a study from Denmark, where high levels of 1,25(OH)_2_D in PHPT-patients were associated with increased bone turnover and decreased BMD [29]. PTH also seemed to influence BMD in distal radius. However, using multiple regression with age, gender, BMI, calcium, phosphate, eGFR, 25(OH)D, 1,25(OH)_2_D, and PTH as covariates, the influence of PTH on BMD in distal radius disappeared, whereas the influence of 1,25(OH)_2_D was still significant. Goltzman et al. showed that the 1,25(OH)_2_D/VDR-system is required for an appropriate osteoclast response to elevated PTH [6]. Both 1,25(OH)_2_D and PTH increase the expression of Receptor Activator NF-κB Ligand (RANKL) from osteoblasts [30], [31]. In turn, RANKL stimulates the maturation of osteoclasts resulting in enhanced bone resorption [31], [32]. Physiological concentrations of 1,25(OH)_2_D inhibit PTH-induced release of RANKL in osteoblasts, while supra-physiological levels of 1,25(OH)_2_D further increase the RANKL-release [30]. This suggests that serum concentrations of 1,25(OH)_2_D above a certain threshold may result in adverse effects on bone [33]. Bone loss in PHPT-patients occurs mainly at sites with high proportion of cortical bone, such as distal radius, while bone loss in trabecular bone, such as lumbar spine, is less prominent [34], [35], [36]. An effect on cortical bone prior to trabecular bone is in line with our findings.

The optimal concentration of vitamin D for beneficial health outcome is still subject for discussion [37], [38]. Based on associations between 25(OH)D, BMD, bone turnover, muscular function and falls, serum levels of 25(OH)D below 50 nmol/L has been characterised as vitamin D inadequacy [39]. However, levels up to 75 nmol/L may still increase the beneficial effects of vitamin D on risk of falls, extremity strength, and prevention of cancer and hypertension [40]. It has been suggested that patients with mild PHPT and vitamin D insufficiency should receive supplementation of vitamin D to ensure 25(OH)D above 50 nmol/L [41], [42]. In a recent study from England, Rao et al reported stable s-Ca levels in addition to a moderate decrease of PTH in patients with PHPT receiving vitamin D supplementation [43]. Furthermore, beneficial effects on bone metabolism with lowered ALP and increased BMD in lumbar spine and femoral neck was observed in vitamin D deficient PHPT patients after vitamin D supplementation [44], [45]. It should be noted that in these studies showing beneficial effects of vitamin D supplementation on PTH levels and bone health, all patients had serum 25(OH)D below 50 nmol/L prior to supplementation [43], [44], [45]. In contrast, supplementation of 1,25(OH)_2_D increased bone turnover in a study on healthy individuals with mean level of 25(OH)D above 65 nmol/L [46]. This is in line with our findings that higher 1,25(OH)_2_D levels seem to have a deleterious effect on BMD in cortical bone. In the present study we did not observe as low levels of 25(OH)D as described in French and Danish studies on PHPT, where more than 80% of the patients had 25(OH)D serum concentrations below 50 nmol/L [8], [9]. In patients with PHPT, 25(OH)D concentrations are found to be a strong determinant for levels of 1,25(OH)_2_D, showing a positive correlation between 25(OH)D and 1,25(OH)_2_D [47]. Initial levels of 25(OH)D and 1,25(OH)_2_D are probably decisive as to whether vitamin D supplementation has beneficial or deleterious skeletal effects.

Both PTH and 1,25(OH)_2_D regulate cytochrome P450 enzymes metabolising vitamin D. SNPs in genes encoding these enzymes are of particular interest in patients with PHPT, where the production of both PTH and 1,25(OH)_2_D is enhanced. According to Wang et al [25], rs10741657 is the locus in the 25-hydroxylase gene with strongest association to 25(OH)D levels. Wang et al also showed an association between 25(OH)D levels and the SNP rs6013897 in the 24-hydroxylase gene. Furthermore, an association with the SNP rs2762939 in the 24-hydroxylase gene and risk of coronary artery calcification was shown. However, this SNP was not correlated to 25(OH)D levels [22]. Rs10877012 in the 1α-hydroxylase gene has also been associated with 25(OH)D levels [21]. In the present study none of these SNPs were associated with levels of 25(OH)D, 1,25(OH)_2_D, PTH, serum calcium or BMD. We observed levels of 1,25(OH)_2_D, the ligand for the VDR, above the reference range in the patients with PHPT. The influence of SNPs in the VDR-gene on bone metabolism could therefore be stronger in patients with PHPT than in a healthy population. Our analyses of the most common SNPs in the VDR revealed an association for the different genotypes of the Apa1 polymorphism (rs7975232) and BMD in the lumbar spine. The minor allele was associated with lower BMD in the lumbar spine. A previous study on osteoporotic women has also shown a relationship between the minor allele of the Apa1 polymorphism and lower BMD in lumbar spine [48]. In twin studies a stronger influence of genetic factors on BMD in the trabecular than the cortical skeleton is observed [49]. This is in accordance with the findings in our study where BMD in lumbar spine, consisting mainly of trabecular bone, but not distal radius, consisting mainly of cortical bone, was influenced by the Apa1 polymorphism. The levels of 1,25(OH)_2_D were not associated with the Apa1 polymorphisms. Thus, it may appear that the effect of the Apa1 polymorphism on BMD in trabecular bone is different from the bone-resorbing effect of 1,25(OH)_2_D on cortical bone. The association between BMD in lumbar spine and the Apa1 polymorphism has to our knowledge not been observed in PHPT patients before. However, further studies are needed to assess if patients with the minor allele of the Apa1 polymorphism have an increased fracture risk and if surgery would be indicated at an earlier disease stage in these patients. Of note, SNPs in the vitamin D binding protein also influence circulating vitamin D levels [25]. Accordingly, these SNPs may be of interest in patients with PHPT and should be evaluated in further studies.

The combined measurement of the vitamin D metabolites 25(OH)D and 1,25(OH)_2_D in the present study increases the understanding of the effects of vitamin D on bone. As the sample size is rather small, the results need to be confirmed in larger follow-up studies. Our findings highlight potential deleterious effects of increased 1,25(OH)_2_D on cortical bone in patients with PHPT. Supplementation with vitamin D in PHPT-patients with low 25(OH)D has been recommended. Randomized, prospective studies are necessary to conclude on safety of vitamin D supplementation and the effect on bone in PHPT.
